# Design and implementation of community engagement interventions towards healthcare quality improvement in Ghana: a methodological approach

**DOI:** 10.1186/s13561-016-0128-0

**Published:** 2016-10-26

**Authors:** Robert Kaba Alhassan, Edward Nketiah-Amponsah, Daniel Kojo Arhinful

**Affiliations:** 1 Amsterdam Institute for Global Health and Development, University of Amsterdam, Amsterdam, The Netherlands; 2 Department of Epidemiology, Noguchi Memorial Institute for Medical Research, University of Ghana, Legon, Accra, Ghana; 3 Department of Economics, University of Ghana, Legon, Accra, Ghana

**Keywords:** Community engagement, Healthcare quality, Primary health facilities, Ghana

## Abstract

**Background:**

Nearly four decades after the Alma-Ata declaration of 1978 on the need for active client/community participation in healthcare, not much has been achieved in this regard particularly in resource constrained countries like Ghana, where over 70 % of communities in rural areas access basic healthcare from primary health facilities. Systematic Community Engagement (SCE) in healthcare quality assessment remains a grey area in many health systems in Africa, albeit the increasing importance in promoting universal access to quality basic healthcare services.

**Purpose/objective:**

Design and implement SCE interventions that involve existing community groups engaged in healthcare quality assessment in 32 intervention primary health facilities.

**Methods:**

The SCE interventions form part of a four year randomized controlled trial (RCT) in the Greater Accra and Western regions of Ghana. Community groups (*n* = 52) were purposively recruited and engaged to assess non-technical components of healthcare quality, recommend quality improvement plans and reward best performing facilities. The interventions comprised of five cyclical implementation steps executed for nearly a year. Wilcoxon sign rank test was used to ascertain differences in group perceptions of service quality during the first and second assessments, and ordered logistic regression analysis performed to determine factors associated with groups’ perception of healthcare quality.

**Results:**

Healthcare quality was perceived to be lowest in non-technical areas such as: information provision to clients, directional signs in clinics, drug availability, fairness in queuing, waiting times, and information provision on use of suggestion boxes and feedback on clients’ complaints. Overall, services in private health facilities were perceived to be better than public facilities (*p* < 0.05). Community groups dominated by artisans and elderly members (60^+^ years) had better perspectives on healthcare quality than youthful groups (Coef. =1.78; 95 % CI = [−0.16 3.72]) and other categories of community groups (Coef. = 0.98; 95 % CI = [−0.10 2.06]).

**Conclusions:**

Non-technical components of healthcare quality remain critical to clients and communities served by primary healthcare providers. The SCE concept is a potential innovative and complementary quality improvement strategy that could help enhance client experiences, trust and confidence in healthcare providers. SCE interventions are more cost effective, community-focused and could easily be scaled-up and sustained by local health authorities.

## Background

Community participation in health service planning and implementation is a key principle in the Alma-Ata Declaration of 1978 with the fourth article stating that “people have the right and duty to participate individually and collectively in the planning and implementation of their health care” [1]. The Declaration also states that primary healthcare should promote maximum community and individual self-reliance and participation in the planning, organisation, operation and control of primary healthcare services. This premise underscores the vital role communities play in the implementation of effective and sustainable health plans and policies. Community engagement in health is essential to developing countries where resources are scarce albeit demand for healthcare remains high. Leveraging existing community structures and resources towards healthcare quality improvement is therefore an option worth considering.

Morgan and Lifshay [2] defined community engagement in the context of public health as dynamic relationships and dialogue between community members and local health professionals with varying degrees of community and higher level health authorities’ involvement in decision-making and control. Coulter [3] indicated that there are at least four roles for community engagement in health namely: determine local needs and aspirations; promote health and reduce health inequalities; improve service design and the quality of healthcare, and strengthen local accountability.

Morgan and Lifshay [2] proposed a framework for structured community engagement summarized into seven (7) steps in descending order: (1)local health authorities taking the lead and directing the community to act; (2)information sharing plan with the community; (3)soliciting periodic community input and consultation; (4)community members serving as conduits of information/feedback to and from the local health authorities; (5)power-sharing that involves defining and solving problems together; (6)community initiating decisions; (7)community sharing information with local health authorities. Though this framework was initially developed in the context of Western healthcare systems with emphasis on chronic diseases, the ideas remain relevant to primary healthcare in developing health systems.

The framework by Morgan and Lifshay [2], the theory of social capital [4] and theory of change [5] informed theoretical basis for the systematic client/community engagement (SCE) interventions described in this paper. Besides these theories, findings of baseline qualitative and quantitative interviews and series of stakeholder engagement workshops informed the design and implementation of the SCE interventions.

Ghana seems to have some level of structured community participation in health service planning and implementation, especially at the primary healthcare level [6–9]. The Community-based Health Planning and Services (CHPS) programme adopted in Ghana in 1999 aimed to reduce barriers to geographical access to healthcare with an initial focus on deprived and remote areas of rural districts [9]. Although the CHPS programme contributed significantly to improved health outcomes and accelerated community participation in health, the focus did not emphasize community engagement in healthcare quality assessment.

In Ghana, client/community involvement in healthcare quality assessment is conventionally limited to exit interviews during patient satisfaction surveys, albeit this strategy is increasingly proving ineffective in healthcare quality improvement. Patient satisfaction surveys (though relevant) have a limitation of potential biased assessment especially when interviews are conducted in health facility premises [10–13]. The limitations of these conventional approaches underscore the need to complement them with structured community engagement in healthcare quality assessment.

This paper describes the methodology, implementation process and outcome of SCE interventions implemented in 32 primary healthcare facilities in two regions in Ghana for nearly 12 months. It is expected that the findings and experiences will guide policy makers and researchers contemplating replication of the SCE interventions in other settings in Africa and beyond.

### Overview of the SCE Interventions

The WOTRO-COHEiSION Ghana project is a four-year randomized controlled trail (RCT) initiated in 2011 to contribute (via evidence-based research findings) towards removing barriers to (re)enrolment in Ghana’s national health insurance scheme through client-centered healthcare and health insurance system [14]. As part of the study design, SCE interventions were implemented using existing community groups/associations to assess healthcare quality in selected primary health facilities.

The SCE interventions aimed at empowering communities in healthcare quality improvement as a strategy to promote client trust and confidence in healthcare providers and the National Health Insurance Scheme (NHIS). The assumption is that active community engagement in healthcare quality assessment has the potential to decrease perceived barriers to utilizing healthcare and health insurance services and ultimately enhance active participation in the NHIS.

Objectives of the SCE interventions include: diminishing identified barriers to enrollment in the NHIS and utilization of healthcare services; increase client/community participation in healthcare quality assessment; reduce communication gaps between clients and healthcare providers through effective information dissemination; increase and sustain provider accountability to clients/communities; empower clients and promote client-centered healthcare and health insurance system in Ghana.

Two categories of SCE interventions were implemented namely: *MyCare* (also called Intensive Engagement) and Light Engagement (LE). The LE intervention used existing community groups/associations to identify gaps in service delivery in healthcare facilities. The identified gaps were communicated to all intervention health facilities and encouraged to initiate necessary corrective measures with a promised token incentive, should service providers succeed in narrowing the quality care gaps.

The *MyCare* component involved clients and relevant stakeholders in a participatory process. The focus was on individual clients contrary to the group approach in the LE. The LE interventions employed mainly qualitative methods while *MyCare* interventions used both qualitative and quantitative methods. Both categories of interventions were implemented and evaluated concurrently. For the purposes of this paper, the emphasis was on the LE interventions. The *MyCare* component of the SCE interventions is detailed in Fenenga et al. [15].

## Methods

### LE interventions setting

**Fig. 1:**
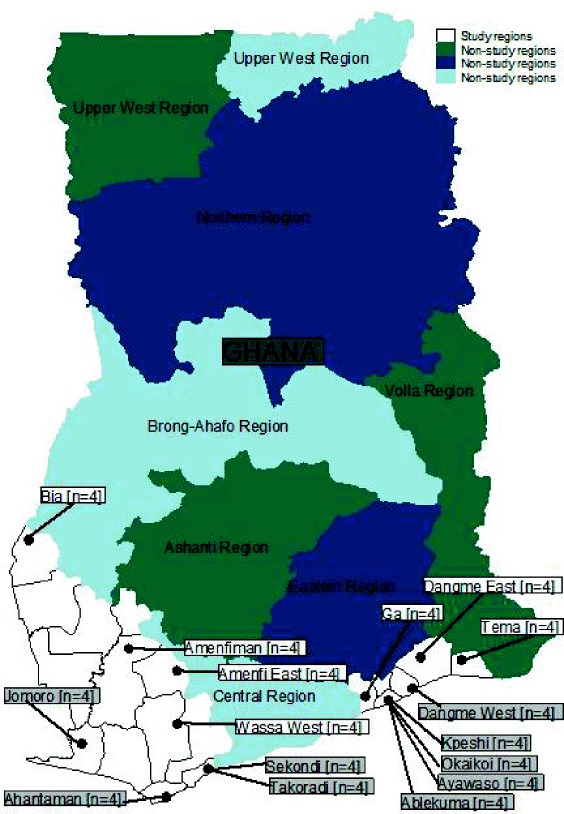
Geographic distribution of study facilities by districts. Source: WOTRO-COHEiSION Ghana Project Health Facility Survey Data (March-June, 2015)

The LE interventions were implemented in Greater Accra (predominantly urban) and Western (predominantly rural) regions of Ghana. Only primary healthcare facilities (i.e. clinics and health centres) accredited by the National Health Insurance Authority (NHIA) were purposively sampled to participate in the study. The NHIA is the regulatory body of the NHIS in Ghana. Accredited clinics/health centres were purposively sampled because they are relatively less complex and could easily be monitored for impact of implemented interventions. A total of 16 administrative districts were sampled at random (eight from each region) and four health facilities allocated to each district. In every district, two out of the four facilities were randomly picked to receive interventions and the remaining two assigned as controls; nine out of the 16 districts were rural and seven were urban. Figure 1 shows geographic distribution of health facilities by districts.

### LE interventions design and randomization

Random allocation of health facilities into the different intervention and control arms of the project was conducted such that in each district, the names of all 4 health facilities were written on pieces of paper. Subsequently, for each district at a time, two ballots (representing health facilities) were randomly picked without replacement to receive intervention. Per this criteria 32 health facilities and their catchment area were randomly assigned as intervention facilities and the remaining 32 as controls.

**Fig. 2:**
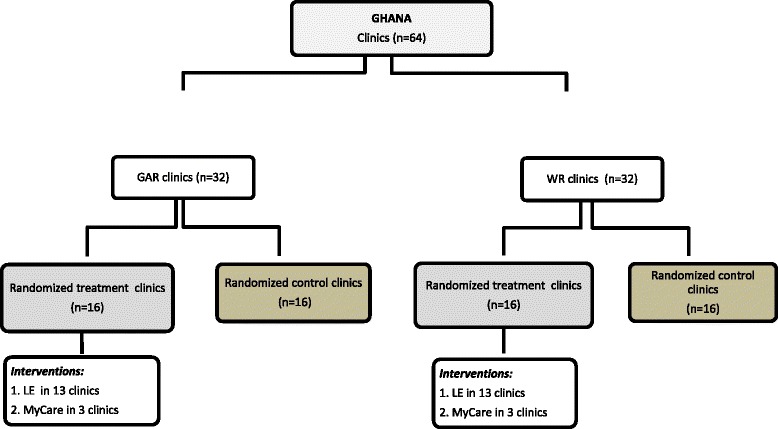
Interventions design. Source: WOTRO-COHEiSION Ghana Project (2013) & Alhassan et al. Perspectives of frontline health workers on Ghana’s National Health Insurance Scheme before and after community engagement interventions. BMC Health Services Research; 2016 16(192): 1–11; Legend: GAR: Greater Accra Region; WR: Western Region; LE: Light Engagement; n=sample size. NOTE: MyCare intervention is detailed in Fenenga et al. (2014) hence it is not elaborated in this paper

Out of the 32 intervention facilities 26 were randomly picked to receive the LE interventions (13 from each region) and the remaining six assigned the *MyCare* interventions (three from each region). Detailed description of the MyCare and LE interventions has been given in Fenenga et al. [15]. In this paper, the focus was on the design and implementation steps of the community engagement interventions and not on impact evaluation of the interventions. In terms of location, 18 of the 32 intervention facilities were sampled from rural areas and 14 from urban areas. In terms of ownership 21 intervention facilities were private and 11 were public. Figure 2 illustrates the interventions design.

### LE implementation steps

The LE interventions comprised of five steps implemented for nearly one year (between June, 2013-March, 2014). The first step involved recruitment and training of facilitators, and identification of existing community groups/associations within the catchment area of the selected health facilities in the sampled districts. A total of 52 facilitators were recruited and trained. One facilitator was assigned to each of the of 52 community groups in the two study regions (26 in each region). Eligibility criteria for selection of community groups included: (i) documented evidence of regular meetings (at least once every two months), (ii) regular meeting venue, (iii) clear leadership structure, (iv) non-partisan in nature and activities and (v) active membership not less than an intuitive number of ten (10). These criteria ensured the groups were active in their activities and reasonably represent a cross-section of community opinion. There was no criteria for selecting individual members of the community groups. Since the focus was on engaging already existing community groups, existing composition of the groups was maintained to reflect the natural situation of the groups.

The second implementation step entailed a first round of community group assessment of healthcare quality based on group members’ most recent experiences with the particular intervention health facility in their community. Healthcare quality proxies used to guide community members during assessment were: (1)staff attitude, (2)punctuality to work, (3)client waiting time, (4)queuing system, (5)availability of drugs, (6)information provision to clients, (7)equal treatment for insured and uninsured clients, (8)complaint system for clients, (9)client-provider communication, and (10)net promoter score (NPS^1^).

During engagement sessions, group members used “Community Score Cards” to rate performance of their nearest health facility on these quality care proxies on a five-point Likert scale from 1 = “Very disappointing” to 5 = “Very Satisfactory”. The group assessments were conducted in the communities to avoid possible bias and client intimidation at the health facility. Group ratings were based on the members’ most recent experiences (at most six months) with the pertinent intervention health facility in the community. Anonymity of group members was assured by reporting group perceptions without individual personal details.

The third implementation step validated community groups’ assessment findings with facility heads, clients and NHIA representatives. This platform provided the service providers the opportunity to recognize and accept gaps in healthcare quality and agree on quality improvement plans with timelines and responsible persons.

**Fig. 3:**
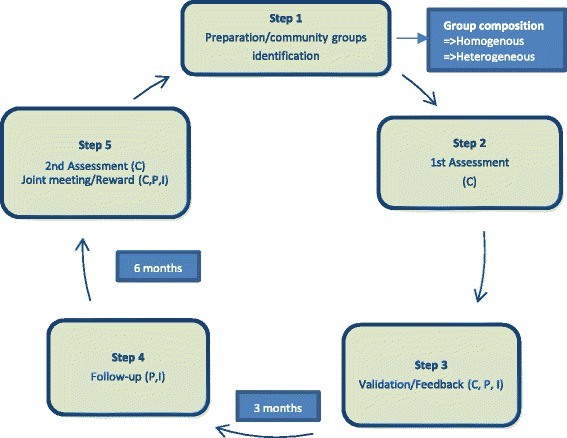
LE implementation steps. Source: WOTRO-COHEiSION Ghana Project (2013) & Alhassan et al. Effect of community engagement interventions on patient safety and risk reduction efforts in primary health facilities: evidence from Ghana. PLoS One; 2015 10(11): 1–19; Legend: C=Client; P=Provider; I=Insurer

During the fourth step, facilitators followed-up on the service providers (three months after validation and feedback sessions) to ascertain whether or not providers were implementing the agreed action plans towards quality improvement. The last step rewarded best performing health facilities after the second round of community assessment (approximately six months after the first assessment). A written citation of honor and a token financial incentive of about US$ 285.0 equivalence in Ghanaian Cedis was awarded to best performing facilities to encourage healthy competition among peers towards quality improvement. Besides the token financial reward given to each best performining health facility, it also cost approximately US$ 100.0^2^ to conduct a round of community engagement session in a community. This amount was used to pay transportation and work allowance of the facilitator. Figure 3 shows the LE engagement implementation steps.

### Ethical considerations

Ethical clearance was sought for the WOTRO-COHEiSION Ghana Project from the Ghana Health Service (GHS) Ethical Review Committee (ERC) (clearance number: GHS-ERC: 18/5/11). Written informed consent was also sought from individual participants who were literates. For those who were illiterates the study protocol was explained to them in the local language before consent was given by thumb-printing.

### Data analysis

Two community groups were required to assess an intervention health facility during the first and second assessments. Per this criterion, total of 52 group reports were retrieved from the two study regions. Though the group assessments were done in the form of group discussions, overall perception per quality care indicator was attained by providing unanimous scores on a five-point Likert scale. This approach yielded quantitative data for analysis.

Group ratings were recorded on the “Community Score Card” and later collated per health facility by trained facilitators. The group responses were all entered into STATA statistical analysis software (version 12.0) after cleaning and coding. Responses were analyzed based on group names and codes. For anonymity purposes, personal details of individual group members were not linked to the quality assessment ratings.

Descriptive statistics were used to analysis the basic socio-demographic characteristics of the groups. Iterated principal factor (IPF) analysis was done to group 12 quality care proxies into five factors using the orthogonal varimax rotation option (Kaiser off). Cronbach’s alpha test was used to determine scale reliability for the 12 Likert scale items and the coefficient was found to be 0.92 which is above the 0.70 rule of thumb [16]. Wilcoxon sign rank test was used to test for group perception differences in the first and second group assessments. Overall differences in group perceptions based on facility ownership, location and region was determined using the Wilcoxon-Mann-Whitney test as appropriate [17, 18]. Factors associated with overall community perception of healthcare quality was determined using the ordered logistic regression (OLR) test at univariate and multivariate levels.

## Results

### Composition of community groups

Out of the 52 engaged community groups, 22 were religious/faith-based; the rest were: eight traders groups; one widows group; three community volunteers groups; three music/singers groups; five artisans groups and 11 youth groups. Average group size during an engagement session was 29 members (Min = 8, Max = 91); thus, about 1,500 community members were engaged during the first and second engagement sessions. More than half of the groups were female dominated; 13 were male dominated; two were all males; five were all females, and one was balanced number of males and females.

Approximately 56 % of the groups were a combination of literates and illiterates; 23 % were mainly literates, and 21 % mainly illiterates. In terms of age, 65 % of the groups had predominantly elderly members (31^+^ years) and 35 % had predominantly youthful members (18–30 years). The average meeting duration per group was 41 min (SD = 13.8) with an average contribution time per participant being 1.4 min (SD = 1.3) (see Table 1).

**Table 1:** Composition of community groups involved in LE interventions

Group characteristics	Average age			Group location		
	18–30 years	31^+^ years		Rural	Urban	
Group type	%	%	*p*-value	%	%	*p*-value
Religious (*n* = 22)	4 %	38 %	0.000**	15 %	27 %	0.432
Traders (*n* = 8)	0 %	15 %		10 %	6 %	
Widows (*n* = 1)	0 %	2 %		2 %	0 %	
CVG (*n* = 3)	8 %	0 %		4 %	2 %	
Music/singers (*n* = 2)	4 %	0 %		2 %	2 %	
Artisans (*n* = 5)	7 %	2 %		8 %	2 %	
Youth groups (*n* = 11)	13 %	7 %		13 %	7 %	
Total (*n* = 52)	36 %	64 %		54 %	46 %	
Gender distribution						
Male dominated (*n* = 15)	17 %	12 %	0.005**	15 %	13 %	0.779
Female dominated (*n* = 36)	17 %	52 %		37 %	33 %	
Equal distribution (*n* = 1)	0 %	2 %		2 %	0 %	
Total	34 %	66 %		54 %	46 %	
Literacy/education						
^+^Mainly literates (*n* = 11)	8 %	13%	0.000**	15 %	6 %	0.421
^++^Mainly illiterates (*n* = 12)	21 %	2%		11 %	12 %	
Literates/illiterates (*n* = 29)	6 %	50%		27 %	29 %	
Total	35 %	65%		53 %	47 %	
Group dynamics	Mean(SD)	Mean(SD)	*p*-value	Mean(SD)	Mean(SD)	*p*-value
Active membership (*n* = 52)	60.4(15.0)	60.2(18.3)	0.9668	56.6(16.9)	64.5(16.6)	0.0967*
Engagement duration (*n* = 52)	41.5(17.0)	40.8(12.1)	0.8634	44.5(15.1)	37.1(11.2)	0.0523*
Average time per discussant (*n* = 52)	1.7(1.4)	1.3(1.3)	0.3139	1.1(1.2)	1.7(1.5)	0.1203

Source: WOTRO-COHEiSION Ghana Project, 2014; Wilcoxon-Mann-Whitney test statistically significant (*p < 0.1; **p < 0.05); ^+^Literates are operationally defined to include those who have a least secondary education certificate; ^++^Illiterates are operationally defined to include those who did not complete basic education or did not attain formal education altogether and cannot read or write

### Community perceptions on healthcare quality

**Fig. 4:**
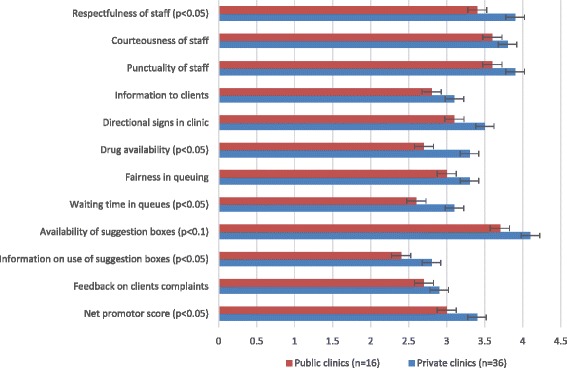
Community perception of healthcare quality graphed by facility ownership. Source: Light Engagement Intervention data of the WOTRO-COHEiSION Ghana Project (2013–2014). Legend: *Mean scores based on the five point Likert from 1 = “Very disappointing” to 5= “Very satisfactory”. High mean score depict higher group satisfaction with pertinent quality care proxy while lower mean scores suggest otherwise. Note: Net promotor score is the chances of recommending the health facility to a friend or relative based on the overall perceived quality of healthcare

Analysis of pooled data of the 52 community group reports showed that information provision to clients on use of suggestion boxes was rated lowest on the five-point Likert scale but private facilities (mean = 2.8, *p* < 0.05) appeared to have been rated higher than public facilities (mean = 2.4, *p* < 0.05). Quality indicators such as client waiting times; drugs availability and respectfulness of health staff were perceived to be relatively better in private than public facilities (*p* < 0.05) (see Fig. 4). Similarly, likelihood of community members recommending their nearest health facility to friends and relatives (net promotor scores) was averagely rated 3.0 for public facilities and 3.4 for private facilities (*p* < 0.05). Likewise, private health facilities were perceived by community members to be relatively better in terms of availability of suggestion boxes for clients; punctuality and courteousness of staff (*p* < 0.05) (see Fig. 4).

Comparison of the first and second group assessment scores showed that perception of healthcare quality appeared to have improved significantly across all the healthcare quality indicators during the second group assessments in 2014, suggesting an improvement over the first assessments conducted in 2013. Community members particularly perceived significant improvement in information provision to clients by healthcare providers (mean diff. = 2.40; *p* < 0.0001). Likewise, perceived drugs availability (mean diff. = 2.27; *p* < 0.0001); provision of feedback on client complaints (mean diff. = 2.02, *p* < 0.0001), and net promotor score (mean diff. = 2.02; *p* < 0.0001) were found to have improved significantly during the second round of assessment. Though there were perceived improvements in staff respectfulness/courteousness towards clients and punctuality to work, the marginal increases were relatively lower (*p* < 0.0001) (see Table 2).

**Table 2:** Mean scores of quality indicators in health facilities (*n* = 52)

	Mean scores			
	2^nd^ assessment (2014)	1^st^ assessment (2013)		*p*-value
Quality indicators	Mean (SD)^b^	Mean (SD)	Mean diff.	
Respectfulness of staff	4.2(0.7)	2.9(1.1)	1.30	0.0000^a^
Courteousness of staff	4.2(0.7)	3.2(1.0)	1.00	0.0000^a^
Punctuality of staff	4.4(0.6)	3.0(1.0)	1.40	0.0000^a^
Clear information provision to clients	4.1(0.8)	1.7(0.8)	2.40	0.0000^a^
Directions to clients in health facility	4.1(0.9)	2.4(1.1)	1.70	0.0000^a^
Availability of drugs	4.0(0.9)	1.8(0.8)	2.20	0.0000^a^
Fair queuing system	3.9(0.8)	2.2(1.2)	1.70	0.0000^a^
Waiting time for clients	3.6(0.9)	1.9(0.9)	1.70	0.0000^a^
Availability of suggestion boxes	4.2(0.7)	3.4(1.1)	0.80	0.0000^a^
Information on use of suggestion boxes	3.4(0.8)	1.52(0.7)	1.88	0.0000^a^
Feedback system on clients’ complaints	3.8(0.9)	1.8(0.8)	2.00	0.0000^a^
Net promotor score^+^	4.2(0.7)	2.2(0.8)	2.00	0.0000^a^
Overall quality score	4.0(0.6)	2.3(0.6)	1.70	0.0000^a^

Source: WOTRO-COHEiSION Ghana Project (2013–2014); ^a^Wilcoxon signed rank test statistically significant (*p* < 0.0001); ^b^Means and SD rounded up to the nearest decimal; ^+^Net promotor score is the client's chances of recommending the health facility to a friend or relative based on the overall perception of healthcare quality in the pertinent health facility

Using the pooled first and second assessments data, it was found (after performing an ordered logistic regression test) that artisan community groups were more likely to rate quality care indicators higher than other types of community groups (Coef. = 1.78; *p* < 0.05) (see Table 3). In terms of age, community groups that were predominantly elderly (31^+^ years) were more likely to perceive healthcare quality more positively than groups with predominantly youthful members (18–30 years) (Coef. =0.98; *p* < 0.05). Table 3 shows other factors associated with overall perceived quality care in the intervention health facilities.

**Table 3:** Factors associated with community groups’ perception of healthcare quality (*n* = 52)

Independent variables	Univariate		Multivariate	
	Coef.	(95 % CI)	Coef.	(95 % CI)
Model 1: Group type				
Religious	1.0	1.0		
Traders	−0.91	(−2.31 0.50)		
Widows	−0.59	(−3.47 2.30)		
CVG	2.03	(−0.66 4.72)		
Music/singers	−0.11	(−2.22 2.00)		
Artisans	1.78*	(−0.16 3.72)		
Youth associations	−0.73	(−2.00 0.55)		
LR chi2(6)	9.27			
Prob > chi2	0.1591			
Pseudo R2	0.0267			
Model 2: Gender distribution				
Equal gender distribution	1.0	1.0		
All male groups	1.86	(−0.77 4.49)		
All female groups	0.92	(−1.41 3.25)		
Male dominated groups	0.61	(−1.53 2.75)		
Female dominated groups	0.16	(−3.29 3.62)		
LR chi2(6)	2.83			
Prob > chi2	0.5872			
Pseudo R2	0.0082			
Model 3: Literacy/education				
Literates/illiterates	1.0	1.0		
Mainly literates	−0.46	(−1.97 1.06)		
Mainly illiterates	0.192	(−1.07 1.46)		
LR chi2(6)	1.10			
Prob > chi2	0.5777			
Pseudo R2	0.0032			
Model 4: Group meetings dynamics				
Active membership			0.01	(−0.02 0.05)
Engagement duration			0.02	(−0.02 0.06)
Average time per discussant			−0.30	(−0.93 0.34)
Age distribution				
Youthful (18–30 years)			Ref	Ref
Elderly (31^+^ years)			0.98*	(−0.10 2.06)
Geographic location				
Urban			Ref	Ref
Rural			0.07	(−1.04 1.17)
LR chi2(6)			7.19	
Prob > chi2			0.2071	
Pseudo R2			0.0207	

Source: WOTRO-COHEiSION Ghana Project (2013–2014); Ordered logistic regression test statistically significant (*p < 0.05). Model fit statistics: Model 1 Log Likelihood = −168.76315; Model 2 Log Likelihood = −171.98312; Model 3 Log Likelihood = −172.8477; Model 4 Log Likelihood = −169.80268

## Discussion

Community engagement in health is not an entirely new concept in Ghana [6, 7, 19, 20] although its implementation and focus has not been on healthcare quality assessment. The CHPS programme in Ghana was one of the key community-based interventions aimed at enhancing community participation in health in line with the Alma-Ata declaration of 1978. Even though the CHPS programme has contributed to increased accessibility to basic healthcare services in Ghana, the programme by design does not appear to include active engagement of community groups in healthcare quality assessment especially in the context of the NHIS. Impact evaluation studies by Alhassan et al. [21–23] on effects of community engagement interventions on healthcare quality, patient safety, efficiency in health service delivery, and health staff experiences with clients corroborate the increasing benefits and perhaps untapped potentials of active community engagement in health service planning and implementation.

In Ghana, healthcare quality assessment and improvement strategies are mainly technical and dominated by medical experts with little or no community engagement in the process. Perhaps this is largely because of the widely held anecdote that healthcare clients lack requisite health information to assess quality healthcare standards. The increasing role of communities and clients in healthcare quality improvement is however becoming evident and compelling for active community engagement to guarantee successful implementation and sustainability of health programmes [6, 8, 21–25].

Although community engagement in healthcare quality assessment has been found to potentially induce high client confidence and trust in health systems [6, 8, 25], the concept is yet to be adequately explored to its full potentials. As demonstrated in this study, effective engagement of communities in quality care improvement could help enhance perceived quality of healthcare and promote good health seeking behavior of clients. This observation is consistent with conclusions in the AU Policy Brief Report [24] and some studies on Ghana [21–23] which intimated that, active involvement of communities in health programmes improves experiences of health staff and clients of service quality in health facilities.

During the SCE interventions, existing community groups were engaged to assess non-technical quality care components in selected health facilities. Perceptions of community groups of service quality was found to be lowest in the areas of client waiting time; client-provider communication; information provision, availability of client complaint systems and respectfulness of staff. These findings are consistent with results of previous studies on Ghana [26, 27] and Burkina Faso [11, 12] where long waiting times, poor staff attitudes towards clients and physical environment of health facilities were identified as major concerns to clients. Unlike this current study, the previous studies [11, 12, 26, 27] were cross-sectional studies that mainly involved healthcare professionals without active community engagement and client participation.

In this study it was found that community groups generally perceived healthcare quality to be better in private than public health facilities. Patient satisfaction surveys conducted in Ghana arrived at similar conclusions stating private facilities are perceived to have better physical work environment, better staff attitudes towards clients and shorter waiting times in queues [27–30]. Even though the healthcare quality assessments were done in groups in this study, the responses nonetheless reflect individual level perceptions and exepriences on service quality in private and public health facilities.

On the whole, community members’ perception scores on healthcare quality improved during the second assessment, suggesting healthcare providers perhaps implemented the recommendations of the community groups towards quality improvement. Particular areas that recorded significant improvement were clear information provision to clients, availability of drugs and complaint systems for clients. Even though the other quality healthcare components also improved, the marginal increases were lower. These findings are consistent with arguments that active involvement of communities is a potential avenue to enhance mutual collaboration between healthcare providers and clients towards quality service improvement [8, 19, 23, 24]. When healthcare providers reckon they are closely monitored by their communities, they are more likely to demonstrate greater accountability to clients [23].

Perception of healthcare quality was found to have an association with the type and composition of the community groups (see Table 3). This observation suggests that group dynamics should be adequately analyzed prior to implementation of the SCE interventions. For instance, community groups that comprise of mainly literates or illiterates could influence their overall judgment of healthcare quality in health facilities. Similarly, gender composition of the community groups was found to have an association with the quality assessment ratings. Groups that composed of only males were more likely to have positive perspectives on healthcare quality (Coef. = 1.86; CI = −0.77 4.49) than groups that were only females (Coef. = 0.92; CI = −1.41 3.25) or dominated by males (Coef. = 0.61; CI = −1.53 2.75) or females (Coef. = 0.16; CI = −3.29 3.32).

In terms of age distribution, it was found that community groups that were predominantly elderly (31^+^ years) were more likely to give a positive assessment of service quality than groups that are relatively younger (18–30 years (Coef. = 0.98, *p* < 0.05). The findings imply age dynamics of community groups should be adequately considered when designing and implementing SCE interventions. On the whole, the findings suggest the need to ensure community groups are reasonably representative of the society to promote sustainability prospects of SCE interventions or similar community engagement interventions.

### Sustainability and scale-up

The SCE interventions are potentially sustainable for resource poor countries in Africa largely because existing local community structures and resources are harnessed for engagement activities. This approach is meant to reduce cost and promote ownership by community members. Moreover, to guarantee sustainability, key stakeholders of Ghana’s healthcare system such as the Ministry of Health (MoH), Ghana Health Service (GHS), NHIA and the district assemblies were actively involved through series of stakeholder workshops preceding the design and implementation of the interventions. Indeed these stakeholder workshops and broad consultations partly informed the design of the interventions. These stakeholders remain critical if the SCE interventions are to be scaled-up nationwide and subsequently adopted as a national quality improvement policy. Participants of the stakeholder workshops provided suggestions on the cadre of community groups to engage and facilitators selected to moderate the community engagement sessions. Furthermore, the stakeholder workshops contributed to development of the “Community Score Card”.

The SCE interventions could easily to be scaled-up by the district health directorates and district NHIA offices which already have existing structures for monitoring healthcare quality in health facilities. The cost effectiveness of the SCE interventions over existing quality improvement strategies has been detailed in Alhassan et al. [21–23]. The SCE interventions are potentially less expensive to implement and could be used to complement existing quality improvement strategies which are predominantly technical with minimal client/community involvement.

To guarantee sustainability, facilitation of the intervention activities was championed by persons who already work with the district health directorates, the NHIS districts offices and district assemblies. This approach guarantees sustainability since these individuals live in the community and understand the peculiar needs of community members. The expectation is that these facilitators can continue to play this role within the monitoring and evaluating (M&E) framework of the GHS and NHIA. On the financial and/or logistical incentives that will be given to best performing facilities, the concept is potentially sustainable since there are existing arrangements by the GHS and NHIA that seek to reward best performing facilities after peer reviews which are already ongoing interventions in Ghana.

Perhaps the NHIA should initiate stakeholder consultations on piloting and possibly scaling up performance-based financing (PBF) where tariff increment and reimbursement packages are directly linked to community-based approach to quality improvement in health facilities willing to render services to NHIS card bearers. As part of the NHIA accreditation procedures, heath facilities could be mandated to conduct predetermined number of community engagement sessions within the year to qualify for accreditation in addition to the mainstream medical technical quality care requirements. This approach will not only encourage commitment to client-centered quality healthcare but more importantly enhance client trust and confidence in accredited health facilities. Provider accountability to clients and communities could also improve once community engagement is entrenched as a pre-requisite for accreditation and NHIS reimbursement.

### Potential challenges in implementation of SCE interventions

First, if the SCE processes are not well monitored and supervised, the interventions might not yield desired outcomes in terms of quality healthcare improvement in health facilities. For instance, homogenous group composition along political or ethnic divisions could result in bias and pre-conceived assessment of service quality which could be inimical to quality improvement efforts in health facilities. In view of this, the district directors of health and the NHIA must be actively involved from the early implementation stages for their support and goodwill in this regard.

In addition, potential client intimidation by healthcare providers could influence data quality. In the light of this, reporting of community group assessments should not include individual personal data identifying names with perceptions on healthcare quality. Likewise, community members should be adequately educated on the tenets of the engagement sessions to avoid personal attacks on individual health staff. This approach will help identify institutional and system level challenges that impede delivery of good quality healthcare services to clients.

Access to vibrant existing community groups could be a challenge to sustainable SCE interventions especially in communities where migration of members (particularly the youth who are vital agents of change) to urban areas has the potential to compromise active group activities. Allowing community facilitators and district health authorities to identify and recruit suitable groups could help control this sustainability threat.

Furthermore, stakeholders at the district level hoping to replicate the intervention activities might need to commit some financial resources to building the capacity of facilitators responsible for coordinating the community engagement activities. Basic training in report writing and community engagement skills will help equip facilitators with the requisite knowledge to guarantee good quality assessment data. Districts that are not well endowed financially might have difficulties creating new budget lines to conduct routine validation workshops as part of the SCE process. In view of this financial constraint, district health directorates could allocate funds from their existing monitoring and supervision budgets to support these validation workshops. Effective community mobilization could also help harness available community resources to support the engagement activities.

Future interventions design and implementation endeavours should consider using larger community groups, perhaps in every region of Ghana, to help improve on the generalizability of the findings. The current design involved only 52 community groups in two out of the ten administrative regions in Ghana. Involvement of many community groups, nationwide, could prove useful in arriving at more concrete conclusions.

Finally, low health literacy among community members, limited numbers of active/organized community groups, health provider apathy over time and political interference are potential sustainability threats to the SCE concept that should be adequately explored and addressed before implementation of SCE interventions.

## Conclusions

Effective and organized engagement of existing community groups and associations for healthcare quality assessment is a potential quality improvement strategy worth adopting by resource constrained countries in Africa. As demonstrated in this paper, SCE interventions are potentially cost effective and could easily be sustained with minimal technical support from local health authorities. Conventional approaches to healthcare quality improvement are increasingly becoming more expensive to sustain yet yield low outcomes in terms of client experiences of healthcare quality. Leveraging existing community resources to support central government’s efforts will not only promote a sense of ownership and acceptability of health policies by community members but also promote sustainability of quality improvement interventions.

Description of the design and implementation of the SCE interventions in this paper is expected to stimulate further scientific discourses on the need to complement existing quality improvement strategies with SCE. Community-based approach to healthcare quality assessment and improvement is critical for promoting client trust and confidence in health providers. Enhanced client confidence in the healthcare system is a necessary recipe for increased participation in health programmes including Ghana’s NHIS. Likewise, increased trust in the healthcare system could contribute to enhanced utilization of safer healthcare services and ultimately improve health outcomes and wellbeing of the population.

## Footnotes

1 Net promotor score (NPS) is an indicator used to determine the possibility of the healthcare client recommending the health facility to a fellow client (e.g., relative, friend or co-worker) based on their personal experiences of the quality of health service delivery. The rating is done on a five-point Likert scale from 1 = “Very disappointing” to 5 =”Very satisfactory”.

2 Approximations are based on an exchange rate of US$1.00 to GHC3.50 (Ghana Cedis) as at March, 2015.
